# The President’s Address

**Published:** 1902-01-15

**Authors:** H. F. Harvey

**Affiliations:** Cleveland, Ohio


### THE PRESIDENT'S ADDRESS.

BY H. F. HARVEY, D.D.S., CLEVELAND, OHIO.

Read at the Thirty-sixth Annual Meeting of the Ohio State Dental Society, Columbus, O., December 3, 1901.

For the objects set forth in its constitution, the Ohio State Dental Society has met for its 36th annual session. The presiding officer, as usual, presents an address at the opening session partaking largely of the suggestion of ideas that might possibly be utilized for the betterment of the society.

I wish to thank the various committees for the work they have done, especially the Tri-State, Executive, Clinic and Committee of Arrangements; for I feel that whatever degree of success may have attended our part at the Tri-State meeting was due to their efforts. In fact I think the success of the entire society depends largely upon the work of its committees. The responsibility of the presiding officer, is therefore, to a very great degree in his appointments. But the committees must have the united support of the members at all times.

I would suggest, first, that the society should appoint standing committees, in the personel of which there should not be more than one change each year, for a committee of three, and not more than two for a committee of five. This gives the committees a chance to work better together.

The work accomplished this year will be summed up in the reports of the committees which will speak of their own successes.

The passage of the army and navy re-organization bill, as enacted, provides for army dental surgeons. In order that we may keep in contact with this new service I suggest the continuation of the committee on army and navy. To support the efforts of the legislative committee to secure a reasonably just law, I would remind you that its work must be assisted by the individual influence of the membership of the society, with the legislative representatives coming from your several districts. And in this connection I would remind the committee of the suggestion made two years ago in reference to exemption of dentists from jury duty.

How to increase the membership is another important question. I would recommend the increase of the membership committee to six, to be appointed in accordance with the previous suggestion in this paper, so that the chairman may remain for a term of years and that this committee be requested to formulate and put into execution some plan of action whereby our membership may be increased.

Although the business and finances of this society might well be left to the faithful care of the Board of Directors, I would suggest that steps be taken toward the establishment of a reserve fund the interest of which in a few years might become sufficient to pay the ordinary expenses of the society; we could then spend more money on our program, securing prominent men to present papers or clinics. Starting this would bring an increase of membership, the fees from which would be sufficient to maintain the society without annual dues or perhaps reduce them greatly. We should endeavor to assist the membership committee with this object in view.

Another point in reference to openings of our sessions. We should adopt some means for bringing about more prompt attendance of all members at the time announced on the program for the opening of each session. We are here for mutual benefit and under considerable expense and all members should see that the time set apart for our sessions should be fully and promptly occupied.

